# Export of evidence-based wellness services: An opportunity to actualize India's demographic dividend

**DOI:** 10.1016/j.jaim.2025.101153

**Published:** 2025-05-22

**Authors:** Rakesh Sarwal, Hemant Bhargav, Badri Narayanan Gopalakrishnan

**Affiliations:** a ESIC Medical College, Faridabad, India; b Integrative Medicine at NIMHANS, Bengaluru, India; c University of Washington Seattle, ISM, United States

**Keywords:** Yoga, AYUSH, Export, Growth, Non-tariff barriers, Cost of healthcare, Wellness, Trade

## Abstract

India's demographic dividend offers a critical opportunity to drive sustained economic growth over the next three decades, driven by increasing its share in global trade. Up until 1700, India had a thriving industrial manufacturing economy, producing about 25 % of the world's industrial output, making it the most important manufacturing centre in international trade. Based on recent trends, we argue that the service sector presents considerable growth potential. Within the service sector, despite a small share currently, the healthcare and wellness demonstrate particular promise. Leveraging India's vast pool of trained professionals and its established expertise in traditional systems of medicine, notably AYUSH (Ayurveda, Yoga and Naturopathy, Unani, Siddha, and Homeopathy), suitable governmental initiatives are needed based on a strategy and a road map to incentivize exporters, get international recognition of Indian certifications, particularly by developed nations. This is essential for the growth of the export of wellness services from India, but can also help reduce the burgeoning healthcare costs, inequity and address the needs of ageing populations in developed countries.

## 1 Introduction

The Prime Minister's vision of India transitioning into a developed nation by 2047, coinciding with the centenary of its independence, is a predication based on the country's demographic potential, resource availability, and a burgeoning youth population [1], [2]. The demographic dividend offers a limited-time opportunity to accelerate economic growth, potentially enabling India to achieve income levels comparable to developed nations. [3]. As India continues to experience robust economic growth, its share in global trade is projected to increase. Concurrently, trade presents a reciprocal opportunity to further expedite India's economic expansion. In this article, we analyse the longitudinal trends in India's trade in goods and services to identify the drivers of promoting export of wellness services using its comparative advantage in systems of traditional medicine.

## 2 Trends in India's trade

**Graph 1:**
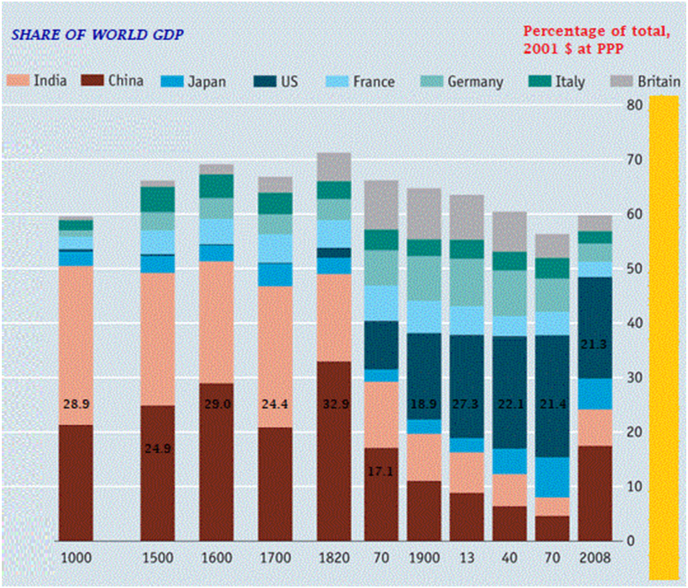
Share of World GDP

Historically, between 1 AD and till 1700 AD, India accounted for 25 % of global GDP, which implicitly reflects an equally significant share in global trade. [4] (Graph-1). This historical data underscores India's substantial economic potential and intrinsic capabilities, supported by its vast resources of land and human capital. Global experience shows that trade raises overall incomes and reduces poverty without necessarily increasing inequality.[5].

In the fiscal year 2022–2023, India's trade, which comprises the export and import of goods and services, accounted for a mere 3 % of global trade,[6] India's trade formed 47 % of its Gross Domestic Product (GDP), in contrast to 60 % of global share of trade to GDP, highlighting a significant gap between India's trade performance and its economic potential.[7] (Table-1). India's Trade Openness Index stands at a mere 17.08, reflecting its limited integration into the global trade system. In contrast trade of Hong Kong, with a Trade Openness Index of 170, constitutes 170 % of its GDP[8]. This disparity underscores the need for India to undertake substantial reforms to enhance its trade openness and increase the share of trade in its economy.

**Table-1:** Trade in goods & services across the World 2023, (billions, US Dollars).

Country	GDP	Population (Billion)	Merchandise Exports	Merchandise Imports	Merchandise Balance	Merchandise trade to GDP	Services Exports	Services Imports	Services Balance	Services to GDP	Trade in services to total trade ( %)	Services trade per capita	Trade to GDP
1	2	3	4	5	6	7	8	9	10	11	12	13	14
India	3568	1.4	432	672	−241	30.9 %	338	246	91	16 %	35 %	406	47 %
BRICS	25938	3.3	4678	3788	891	32.6 %	826	977	−151	7 %	18 %	555	40 %
OECD	64747	1.4	13328	14526	−1198	43.0 %	5509	4826	682	16 %	27 %	7458	59 %
World	106171	8.1	23813	24255	−441	45.3 %	7913	7341	572	14 %	24 %	1893	60 %
India to World	3.36 %	17.84 %	1.81 %	2.77 %	2.30 %		4.27 %	3.36 %	3.83 %				

Strategic policies aimed at improving trade infrastructure, diversifying the export basket, and fostering trade agreements could facilitate India's integration into global markets and enhance its trade competitiveness.[9] Addressing barriers to trade, improving infrastructure, and fostering a conducive regulatory environment are crucial steps for India to enhance its global trade participation and capitalize on its economic potential.[7]

## 3 An opportunity in trade in services

The services share of global trade has increased from just 9 % in 1970 to 24 % in 2023. While global merchandise trade grew by 12 % in 2022, trade in services experienced a more robust growth of 15 % [7] Trade in services is expected to rise to one-third of global trade by 2040, based on a World Trade Organization (WTO) forecast.[10]

The services sector is contributing almost three-fifths to global GDP and about half of the employment opportunities across the world.[11] As a share of GDP, India's trade in services at 16 % is higher than 14 % globally[8] (Table-1)

**Graph 2:**
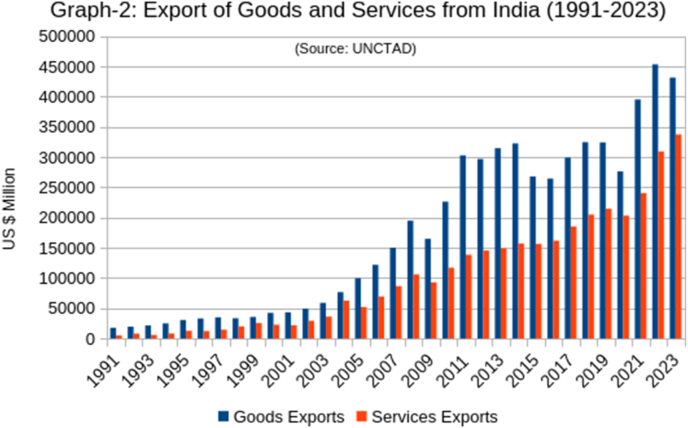
Export of goods and services from India (1991-2023).

**Graph 3:**
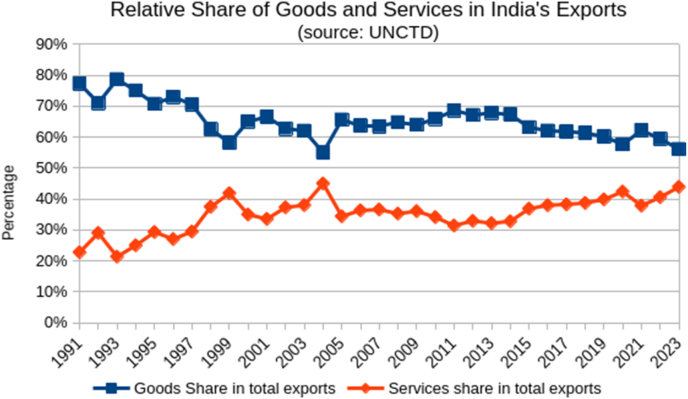
Relative Share of goods and servcices in India'a exports.

Our analysis using data from UNCTAD[8] reveals that the share of services exports in total exports from India has grown from a mere 23 % in 1991 to 44 % in 2023. In contrast, the share of goods in total exports from India has declined from 77 % in 1991 to 56 % in 2023–24?????????????? (Graph 2, Graph 3), Notably, India's export of services during last five years (2018–23) have been growing at twice the rate for goods (Graph-2). Furthermore, India's share in global export of services (4.2 %) is more than double that of its share in overall exports (1.81 %, Table-1). While trade in services makes up 24 % of global trade, it comprises 35 % of India's total trade, highlighting the sector's significance in the Indian economy. (Tale-1) Additionally, India enjoys a net surplus in its balance of trade in services, amounting to $91 billion, contrasting sharply with a deficit of $241 billion in trade in goods.

A distinguishing feature of the services exports from India, in contrast to export of goods, has been its robustness, even during Covid-19 times. (standard Deviations of 92 and 140 Billion US$ respectively during 1991–2023) This acceleration positions India as a key player in the global services market, reflecting its competitive advantage in sectors such as information technology, business process outsourcing, and healthcare services.

India is currently the second-largest service exporter among developing economies, following China. In 2023, India's service exports were less than 73 % of China's and only a third of the United States' exports in services [8]. When considering the human-dependent nature of export in services, it is insightful to compare service exports on a per capita basis; India's service exports per capita stood at $406 in 2023, while the global average was $1893 (Table-1). Currently, India's services exports form a quarter of its share of the global population (4.3 % vs. 17.8 %). This disparity indicates a substantial potential for India to expand its trade in services by leveraging its unique strengths in human dependent sectors such as information technology, healthcare, and traditional medicine. By strategically investing in these areas and enhancing global outreach, India can significantly increase its contribution to global services trade.

## 4 Limits to growth of merchandise export from India

UNCTAD measures nations productive capacities as “the productive resources, entrepreneurial capabilities and production linkages, which together determine the capacity of a country to produce goods and services and enable it to grow and develop.” India's rank of 108 globally on Productive Capacities Index indicates a moderate productive capacity, suggesting constraints on the immediate increase in merchandise exports [12]. Eight out of ten most polluted cities globally are located in India, a rise from six in the previous year [13], posing a significant challenges to a jump in manufacturing growth. A substantial leap in India's manufacturing capabilities include limited availability of land given the pressure of population, inadequate infrastructure, and constraints in transportation and port facilities.[14]

## 5 Limited diversity in current export of services

A significant portion of global trade in services is dominated by the transport and travel sectors, the balance coming from insurance and pension services, financial services, and telecommunications, computer, and information services. In 2023, India's total services exports reached $338 billion, with a substantial contribution from the hospitality and tourism sectors. The value of digitally deliverable services, including IT and IT-enabled services, saw an increase during the COVID-19 pandemic, underscoring their growing importance in the global market. An emerging area with the potential to overshadow traditional sectors is personal, cultural, and recreational services, where India has a historical advantage. Additionally, business services such as engineering, architecture, legal, and accounting services, along with research and management consulting, stand to benefit significantly from government initiatives aimed at enhancing competitiveness in these sectors. [15] The international trade of health services is known to generate positive impacts both globally and locally, fostering economic growth and improving access to essential health services [16]. Yet, the contribution of medical services, particularly those related to AYUSH, is often clubbed with tourism, and not separately reported.

## 6 Role of AYUSH in evidence based wellness industry

Wellness is defined as the active pursuit of activities, choices, and lifestyles that lead to a state of holistic health. [17] Wellness economy is measured in eleven sectors, which include Healthy Eating, Nutrition, & Weight Loss, Physical Activity, Traditional & Complementary Medicine, Prevention, & Personalized Medicine, Mental Wellness and Workplace Wellness. The global wellness market, valued at over $4.5 trillion, is experiencing rapid growth, with countries such as the United States and China leading in wellness tourism and holistic health services[17].

India holds an 18 % share of the global medical tourism market, with approximately 697,453 foreign tourists visiting for medical treatment in 2019, constituting 6.4 % of total foreign tourist arrivals in the country. India's wellness sector is projected to grow at a compound annual growth rate (CAGR) of 18 %, driven by increasing domestic and international demand for Ayurveda, yoga, and natural therapies [18]. According to the Services Export Promotion Council of India [11], the hospital industry in India is projected to grow from Rs. 4 trillion (US$ 61.79 billion) in FY2017 to Rs. 8.6 trillion (US$ 132.84 billion) by FY2022, reflecting a compound annual growth rate (CAGR) of 16–17 %. As one of the largest sectors in India in terms of both revenue and employment, the healthcare industry possesses significant growth potential, driven by strong human resources, an influx of foreign investments, innovations, and advanced technologies.

AYUSH disciplines are build on traditional wisdom, have been usefully practiced over generations which are now being validated by modern scientific methods. Ayurveda, Yoga together have over 17K research articles on PubMed. With Integrative Medicine been accepted as a super speciality in the west, the frontiers of scientifically validating traditional remedies are ever expanding. India recognizes the six disciplines of AYUSH as a part of the legal medical system. It has a large pool of scientifically trained AYUSH practitioners, who are rendering yeoman service in primary and preventive care.[19] There are around 8 Lakh qualified and registered AYUSH practitioners in India.[20] A data base listing fundamental and clinical research in each of the AYUSH sysstems has been developed (https://ayushportal.nic.in/)

As consumers increasingly seek alternative and complementary healthcare options, the AYUSH system, which encompasses Ayurveda, Yoga, Unani, Siddha, and Homeopathy, has gained prominence for its evidence-based practices and natural remedies, which emphasize mental, physical, and spiritual well-being[21]. For instance, Ayurveda advocates personalized healthcare through herbal formulations and lifestyle modifications, while Yoga enhances mental resilience and physical fitness [22] Moreover, the integration of AYUSH therapies into mainstream healthcare has been encouraged by government initiatives in India, recognizing their potential to alleviate the burden of lifestyle-related diseases[23]. This holistic approach not only addresses health concerns but also fosters a preventive mindset, aligning well with the contemporary emphasis on wellness and quality of life. Thus, AYUSH system can can play a pivotal role in the global wellness industry by promoting evidence based, holistic health and preventive care.

However, to fully capitalize on this potential, India must enhance its global marketing strategies, improve regulatory frameworks, and invest in infrastructure to attract wellness tourists and practitioners from around the world. By addressing these challenges, India can position itself as a leader in the burgeoning global wellness market, similar to the success seen in countries like Thailand, known for their wellness tourism initiatives [24]

## 7 Efforts by the government of India in capitalizing wellness services with special focus on AYUSH

The Government of India has implemented various initiatives aimed at promoting service exports, particularly in the wellness sector, which includes AYUSH. Medical Value Travel Services are one of the 12 identified Champion Services. [11]With the establishment of the Ayush Ministry in 2014, there has been a focus on the development and promotion of traditional medicine systems in India and abroad. Another notable initiative was the Service Exports from India Scheme (SEIS), which provided incentives on the net foreign exchange earned by eligible entities in India. This scheme included medical and dental services, hospital services, and health-related and social services, including those provided by midwives, nurses, physiotherapists, and paramedical personnel[25] However, the SEIS was discontinued in 2023 due to resource constraints. In its final year of operation (2022–23), the SEIS awarded duty credits worth Rs. 2455 crore, correlating to exports valued at Rs. 49,100 crore (approximately US $6.13 billion), which represented a cost of merely 2 % of India's total service exports [25].

In addition to SEIS, the government launched the Market Access Initiative Scheme in 2021 (MAI Scheme) to serve as a catalyst for promoting India's exports of goods and services sustainably. This initiative specifically aims to promote traditional Indian products and services, including AYUSH and yoga [9]. Furthermore, the government has engaged in international collaborations and partnerships to promote AYUSH globally. Initiatives such as the Global AYUSH Investment and Innovation Summit have been organized to attract foreign investment and facilitate knowledge exchange in AYUSH practices. These efforts aim not only to enhance the global visibility of India's wellness services but also to integrate AYUSH into global health systems. Recently, the WHO Global Traditional Medicine Centre (WHO- GTMC) was established at Jamnagar, Gujarat. An AYUSH export promotion council was established in 2022.

## 8 Addressing global healthcare needs

Burgeoning cost of healthcare in developed countries, particularly in the United States, has highlighted the urgent need for reforms in the accreditation and practice systems for healthcare professionals globally, aiming to align the demand for high-quality providers with the available supply. [26] In contrast, the cost of healthcare services in India remains significantly lower; for example, an average private consultation in India costs approximately $31, which is about a quarter of the cost of similar services in the United States[27]. This disparity underscores the potential for India to contribute to global healthcare needs by leveraging its qualified healthcare professionals and more affordable services.

For the growth of healthcare and culture-related services, it is essential to lower the non-tariff barriers in western countries and facilitate a more equitable movement of professionals across the globe. Health and social services get to garner one of the lowest levels of commitments in successive trade negotiations, limiting potential growth and collaboration[28]. With the advancement of tele-medicine, which enables the provision of medical services remotely, the cross-border supply of services is becoming increasingly critical. This shift not only enhances accessibility to healthcare but also promotes international cooperation in health-related fields. Health is one of the digitally deliverable services under UNCTAD's classification. Developing countries are doing particularly well in digitally deliverable services, contributing to more than three quarters of exports of these services.[8]

India needs to develop a strategy and a road map to plan for the expansion of opportunities in the export of wellness services, both digitally through tele-medicine, and in person through global placement of its experts and offering high quality, end-to-end flexible tourism and residence packages in the country.

## 9 Challenges in global acceptance of indian health certifications and the need for advocacy in AYUSH

India's National Product Classification for Services Sector, issued by the Central Statistical Organization (CSO), specifies codes for AYUSH systems under human health services under Group "9993199"[29]. This classification facilitates the tracking and reporting of services within the country, promoting better understanding and development of the AYUSH sector. In contrast, the International Trade Classification for Health Services (ITCHS) used globally lacks specific codes for AYUSH services, which hampers the ability to assess and promote these services in international markets. The establishment of inclusive international trade codes for AYUSH services would necessitate international collaboration and consensus among countries, highlighting the need for global efforts to recognize and integrate traditional wellness practices into the global trade framework.

An absolute constraint in the provision of healthcare services from India is the acceptance of Indian certifications by global licensing authorities, particularly those based in the United States, which regulate the practice of these professions in their countries.

To fully capitalize on India's potential for export of wellness services, establishing a global skill recognition regime for Indian qualifications, particularly in the health and wellness sector, is imperative. India has an organized and codified system of traditional knowledge in AYUSH with regulations for teaching, certification and practice in place. These systems of traditional medicine emphasize prevention and lifestyle practices that are particularly effective in managing chronic, non-communicable, or degenerative health conditions. Most of these therapies can be delivered remotely through tele-medicine. Yet, the qualifications awarded by Indian institutes and bodies (such as the Yoga Certification Board) are not recognized beyond India's borders, limiting the export of these services. Paradoxically, accreditation bodies certifying credentials in traditional Indian systems have emerged in the U.S. and are rapidly gained global acceptance. Organizations such as the Yoga Alliance (https://yogaalliance.org/), the International Association of Yoga Therapists (https://www.iayt.org/), and the Ayurvedic Accreditation Commission (https://www.ayurvedicaccreditation.org/) have seemingly appropriated traditional knowledge from India, generating significant income while excluding India's classical teaching centers. In modern terms, such usage without proper attribution could be labeled as theft of Intellectual Property. India should raise this issue in trade negotiations to secure its rightful place in the governance system for the accreditation of professional qualifications in disciplines like yoga and Ayurveda.

UNCTAD and WHO had jointly declared in a statement in 1998 the importance of developing internationally comparable professional standards or advancing their mutual recognition for promoting trade in services. Further liberalization of trade in this area could call for a new approach to negotiations on the movement of natural persons in general, and development of the overall multilateral framework agreement for the temporary movement of natural persons. [30] Such a framework is essential not only to enhance global service exports but also to address the growing healthcare needs, especially of the ageing populations worldwide.[31], [32]

## 10 Conclusion

In conclusion, India's demographic dividend offers a significant opportunity to enhance its position in global trade, particularly within the wellness services sector through the evidence based, AYUSH framework. While government initiatives like the Service Exports from India Scheme and the Market Access Initiative demonstrate commitment, further reforms are essential to overcome barriers such as the lack of global recognition for Indian health certifications and non-tariff trade restrictions.

Given India's inherent strengths in traditional knowledge, rich cultural heritage, and skilled human resource, it has a "comparative advantage" in the export of wellness services. By capitalizing on its strengths in traditional medicine, wellness practices, and healthcare innovation, India can enhance its position in the global services market and contribute to the overall well-being of its population and the world.

By advocating for the inclusion of AYUSH services in international trade classifications and leveraging the cost-effectiveness of its healthcare offerings, India can address global healthcare needs while boosting its economy. A focused effort to enhance the visibility and credibility of traditional Indian practices will not only benefit India economically but also contribute to an evidence based approach to global practice of traditional medicine for wellness.

## Conflict of interest

The authors declare that they have no known competing financial interests or personal relationships that could have appeared to influence the work reported in this paper

## Funding sources

None

## Author contributions

RS conceived the study, and wrote the first draft. HB refined the draft and lead in responding to reviewers comments. BN worked on data sources and provided the table.

## Declaration of generative AI in scientific writing

Not used.
